# Cul4B regulates neural progenitor cell growth

**DOI:** 10.1186/1471-2202-13-112

**Published:** 2012-09-19

**Authors:** Helio C Liu, Grigori Enikolopov, Yuzhi Chen

**Affiliations:** 1 Department of Geriatrics, University of Arkansas for Medical Sciences, Slot 807, Little Rock, AR, 72205, USA; 2 Department of Neurobiology & Developmental Sciences, University of Arkansas for Medical Sciences, Little Rock, AR, 72205, USA; 3 Cold Spring Harbor Laboratory, 1 Bungtown Road, Cold Spring Harbor, NY, 11724, USA

**Keywords:** Cullin, Neurogenesis, Ubiquitination, Neddylation, Mental retardation, β-catenin

## Abstract

**Background:**

Cullin ubiquitin ligases are activated via the covalent modification of Cullins by the small ubiquitin-like protein nedd8 in a process called neddylation. Genetic mutations of *cullin-4b (cul4b)* cause a prevalent type of X-linked intellectual disability (XLID) in males, but the physiological function of Cul4B in neuronal cells remains unclear.

**Results:**

There are three major isoforms of Cul4B (1, 2, and 3) in human and rodent tissues. By examining the endogenous Cul4B isoforms in the brain, this study demonstrates that Cul4B-1 and Cul4B-2 isoforms are unneddylated and more abundant in the brain whereas the lesser species Cul4B-3 that misses the N-terminus present in the other two isoforms is neddylated. The data suggest that the N-terminus of Cul4B inhibits neddylation in the larger isoforms. Immunostaining of human NT-2 cells also shows that most Cul4B is unneddylated, especially when it is localized in the process in G0-synchronized cells. This study demonstrates that Cul4B accumulates during mitosis and downregulation of Cul4B arrests NPCs and NT-2 cells in the G2/M phase of the cell cycle. In both human and rodent brain tissues, Cul4B-positive cells accumulate β-catenin in the dentate subgranular zone and the subventricular zone. These Cul4B-positive cells also co-express the MPM-2 mitotic epitope, suggesting that Cul4B is also necessary for mitosis progression *in vivo*.

**Conclusions:**

This study provides first evidence that unneddylated Cul4B isoforms exist in the brain and are necessary for mitosis progression in NPCs. The data suggest that unneddylated Cul4B isoforms specifically inhibits β-catenin degradation during mitosis. Furthermore, unneddylated Cul4B may play a role in addition to cell cycle since it is exclusively localized to the processes in starved NT-2 cells. Further analyses of the different isoforms of Cul4B will help understand the cognitive deficits in Cul4B-linked XLID and give insights into drug and biomarker discoveries.

## Background

Ubiquitin ligases control the modification of proteins with ubiquitin by interacting with specific substrates. They play critical roles in neuronal functions and homeostasis. For example, mutations of the ubiquitin ligase Ube3A cause Angelman syndrome [1,2]. Ube3A regulates the degradation of Arc, a synaptic protein that promotes the internalization of the AMPA subtype of glutamate receptors [3]. Another example is that the ubiquitin ligase Parkin is mutated in early onset Parkinson’s disease [4]. Parkin is involved in the clearance of damaged mitochondria [5]. It has been shown recently that mutations of another ubiquitin ligase *cullin-4b* (*cul4b)* in the X chromosome cause X-linked intellectual disability (XLID) in males [6-8]. Genetic mutations of *cul4b* account for about 3% of the XLID population, which places *cul4b* as one of the most commonly mutated genes underlying XLID [8]. Such *cul4b* mutations include large C-terminal truncations and missense mutations in or near the Cullin domain that may impair or nullify Cul4B ubiquitin ligase activity [7,8]. Intellectual disability is a consistent feature associated with Cul4B-XLID, but the physiological function of Cul4B in neuronal cells or neurodevelopment remains to be determined.

Cul4B belongs to the family of Cullin-RING ubiquitin ligases (CRLs), in which the Cullin serves as the scaffolding protein in the modular CRLs [9]. Cullins are covalently modified by the ubiquitin-like protein nedd8 in a conserved lysine residue in the C-terminus that also binds to a RING (*r*eally *in*teresting *g*ene) protein Rbx1 or Rbx2 [10,11]. Neddylation of Cullins activates CRLs by inducing a conformational change that promotes ubiquitin transfer to the substrate for ubiquitination [12-14]. The N-terminus of Cullins interacts with Cullin-specific substrate receptor subunits usually via adaptor proteins [9,15]. Cullin neddylation depends on the assembly of the CRL complex including the RING protein and the substrates. Besides neddylation that activates CRLs, the COP9 signalosome (COP9) and CAND1 also positively regulate CRL activity [15-17]. Another regulator ASPP2 inhibits Cullin neddylation and opposes APP-BP1-mediated cell cycle progression [10,18].

Among the seven Cullins identified, Cul4A and Cul4B are derived from the same ancestral Cul4. There is 83% identity between human Cul4A and Cul4B. Cul4B is present only in mammals and differs from Cul4A mainly by an amino terminal extension that may confer Cul4B-specificity. The architecture of Cul4s looks similar, with both binding to Rbx1 and the adaptor protein DNA damage-binding protein-1 (DDB1) [19]. DDB1 binds to a substrate-specific WD-repeat-containing protein [19]. Cul4A is necessary for embryonic development since Cul4A knockout mice are embryonic lethal [20]. However, the lethality was questioned since a second line of Cul4A mutant mice that delete exons 17-19 that encode the Rbx1 binding site and the neddylation site in the *cul4a* gene, produces viable mice due. The lethality of the first strain may be caused by the unintentional deletion of a region upstream of the first exon of the essential *Psid2* gene in the Cul4A mutant mice [21].

The functional analyses of Cul4B are hindered by the lack of a knockout animal model, but available data in literature suggest that Cul4B plays significant roles in many aspects of cellular functions. Like Cul4A, Cul4B also ubiquitinates histone H2A, H3, and H4, facilitating the recruitment of repair proteins to the damaged DNA [22,23]. Cul4B mutant carrier-derived cells are impaired in camptothecin-induced topoisomerase I degradation and ubiquitination [24]. Cul4B has also been shown to regulate dioxin-dependent receptor signaling [25]. In addition, Cul4B downregulates cyclin E [26,27] and β-catenin [28,29]. Cul4B suppression increases β-catenin levels in rodent cells [29]. Cul4B accumulates in the nucleus during cell differentiation [28]. More recently, Cul4B has been shown to downregulate WDR5 [30] and Peroxiredoxin III [31].

Motivated by the recent discoveries that Cul4B mutations cause XLID, we analyzed Cul4B functions in neuronal tissues *in vivo* and *in vitro*. We found that unneddylated Cul4B isoforms accumulated during mitosis of the cell cycle in neural progenitor cells (NPCs). Downregulation of Cul4B by shRNAs arrested the cell cycle in the G2/M phase in NPCs and in human NT-2 cells. NPCs that strongly expressed Cul4B were found in the subventricular zone (SVZ) and the subgranular zone (SGZ) in both mouse and human brain tissues. Such Cul4B-positive cells co-expressed high levels of β-catenin *in vivo*. Furthermore, these Cul4B-expressing NPCs were not apoptotic based on TUNEL staining and flow cytometric analyses. Taken together, these data suggest that the accumulation of unneddylated Cul4B is necessary for mitosis progression in NPCs.

## Results

### Cul4B expression in rodent brain extracts by immunoprecipitation-western blotting

**Figure 1:**
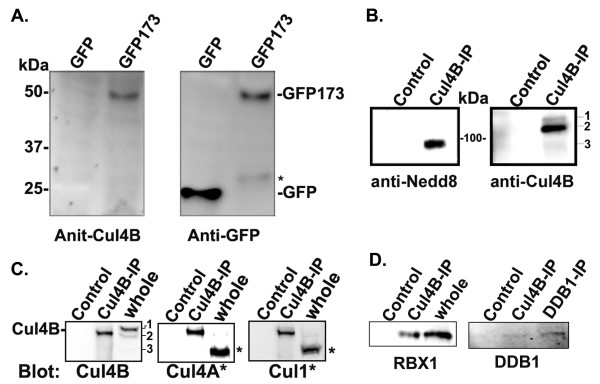
**The anti-Cul4B antibody mainly recognized the larger, unneddylated Cul4B isoforms, Cul4B-1 and Cul4B-2.A**. Cul4B antibody specifically recognized a Cul4B fusion protein. Protein extracts from NPCs transfected with a construct consisting of Cul4B N-terminal 173 amino acids fused to GFP (GFP173). *Left,* The anti-Cul4B antibody recognized the Cul4B fragment GFP173. *Right,* GFP173 was confirmed by the anti-GFP blot. The * indicates the band of cleaved N-terminal GFP173.**B**. Analyses of Cul4B neddylation by immunoprecipitation-western using mouse brain extracts*.* Cul4B was immunoprecipiated with the rabbit anti-Cul4B antibody prebound to protein A-agarose. The negative control was immunoprecipitation with protein A-agarose without antibodies. *Left,* The blot was first probed with a rabbit anti-nedd8 antibody. A smaller band corresponding to the size of Cul4B-3 was neddylated. *Right,* The blot was stripped and reprobed with the Cul4B antibody. The major band corresponds to the MW of Cul4B-2. A minor band of higher MW corresponds to Cul4B-1. Cul4B-3 is also vaguely present. *n = 3.***C**. The Cul4B antibody did not recognize Cul4A or Cul1. Cul4B was immunoprecipitated from mouse brain protein extracts as in B in triplicates. The blots were blotted with the anti-Cul4B, anti-Cul4A, or anti-Cul1 antibodies. The results show that the neddylated band in B was not Cul4A or Cul1. The results also showed that the Cul1 and Cul4A antibodies cross-reacted with Cul4B-2. The Cul4A and Cul1 bands in C are indicated by a *.**D**. The anti-Cul4B precipitates RBX1 in NT-2 cell extracts (*n = 2*) and DDB1 in mouse brain extracts. (*n = 3*).

The rabbit anti-Cul4B antibody obtained from Proteintech was raised against Cul4B(1-350), which consisted of Cul4A homologous sequences that might lead to cross-reactions with Cul4A (personal communications with Proteintech). To determine the specificity of this anti-Cul4B antibody, NPCs were transfected with GFP or GFP-tagged N-terminal 173 amino acids of Cul4B (GFP173). The whole cell lysate was probed with either the anti-Cul4B or mouse anti-GFP. As shown in Figure 1A, the Cul4B antibody specifically recognized GFP173 (*left*), whereas the anti-GFP antibody recognized both GFP173 and GFP (*right*). A minor band around 60 amino-acid longer than GFP was also found in the GFP173-expressing cells as indicated by a *, which suggests that Cul4B might be cleaved near the nuclear localization signal on the N-terminus [27].

The C-terminal Cul4B has the consensus neddylation site and has been shown to be neddylated *in vitro*[32,33]. Neddylation of Cullins may serve as a switch for a conformational change that leads to the activation of ubiquitination by CRLs. To determine if Cul4B was neddylated, adult mouse brain protein extracts were immunoprecipitated with the anti-Cul4B antibody. The blot was first probed with the anti-nedd8 antibody. Rat and mouse Cul4Bs have three major isoforms due to alternative splicing based on the sequences from the UniProtKB/Swiss-Prot data base. The calculated molecular weights (MWs) of mouse Cul4Bs were 111 (Cul4B-1), 107 (Cul4B-2), and 87 kDa (Cul4B-3). Compared to Cul4B-1, Cul4B-3 missed the first 187 amino acids whereas Cul4B-2 missed amino acids 693-721. As shown in Figure 1B (*left*), the band recognized by the anti-nedd8 antibody was likely to be Cul4B-3. The rabbit anti-nedd8 antibody used in the blots was the best anti-nedd8 and is commonly used by other researchers.

To determine if the anti-Cul4B immunoprecipitation experiment worked, the blot was stripped and reprobed with the anti-Cul4B antibody. The major immunoprecipitated band corresponded to Cul4B-2 since it migrated at the predicted molecular weight (Figure 1B, *right*). A minor upper band corresponding to Cul4B-1 was also detected by the Cul4B antibody. However, as shown by the nedd8 blot, Cul4B-1 and -2 were not neddylated since the anti-nedd8 antibody did not recognized these bands (Figure 1B, *left*).

The nedd8-positive lower band precipitated by the anti-Cul4B antibody was further evaluated with antibodies against Cul4A and Cul1. In this experiment, the immuno-precipitates were analyzed in triplicates by blotting with the anti-Cul4B, anti-Cul4A, and anti-Cul1 antibodies, respectively (Figure 1C). The results showed that the anti-Cul4A or anti-Cul1 antibodies recognized a band corresponding to the molecular weights of Cul4A and Cul1, respectively, in the whole brain extract, but they did not recognize the neddylated lower band in the Cul4B precipitate as identified in Figure 1B (*left*). These experiments also showed that the anti-Cul4A and anti-Cul1 antibodies cross-reacted with Cul4B since they recognized the immunoprecipitated Cul4B-2. The results suggest that the lower neddylated band identified in Figure 1B (left) was not Cul4A or Cul1. Thus, the neddylated band was likely to be Cul4B-3. Cul4B-1 and Cul4B-2 appeared to be the major species in the brain as shown in the whole brain extract, but the antibody precipitated more Cul4B-2. Cul4B-3 might be the least abundant since the anti-Cul4B antibody did not recognize this isoform in the whole brain extract. However, the efficiency of the anti-Cul4B in recognizing Cul4B-3 might be less since this isoform missed the first 187 amino acids of Cul4B-1 and 2, which was part of the antigen. Nonetheless, these data demonstrated that the anti-Cul4B in use mainly recognized Cul4B, especially Cul4B-1 and Cul4B-2, but not other Cullins. The data also showed that the larger Cul4B isoforms were unneddylated.

We next examined if the anti-Cul4B antibody would also bring down the ligase adaptors. The antibody precipitated RBX1 in NT-2 cells and DDB1 from mouse brain homogenate (Figure 1D). RBX1 was very abundant in NT-2 cells. Since the level of DDB1 in whole brain lysate was below the detection limit, the anti-DDB1 immunoprecipitate was used as a positive control. DDB1 appeared to be a limiting factor and the amount co-precipitated by the Cul4B antibody was less than that precipitated by the DDB1 antibody, suggesting that only some Cul4B (probably only Cul4B-3) was bound to DDB1.

### Cul4B was not neddylated *in vitro* or *in vivo* as shown by immunostaining

**Figure 2:**
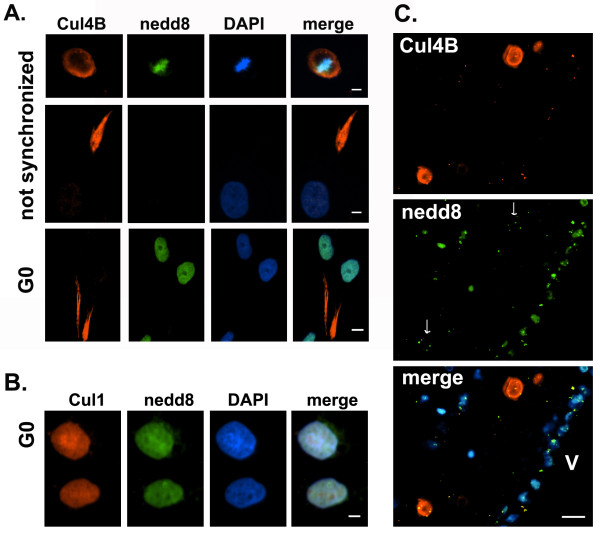
**Cul4B did not colocalize with nedd8. A**. Cul4Bs were not neddylated in NT-2 cells. The top row shows a metaphase cell in which most Cul4Bs did not co-localize with nedd8. The middle role shows a cell from an old NT-2 that had a Cul4B-positive process where Cul4B was not neddylated. The bottom row shows G0-synchronized NT-2 cells in which nedd8 was induced by synchronization in 0.02% FBS for 48 h. These cells also had unneddylated Cul4Bs in processes. *The space bars represent 5, 5, and 10 μm, respectively.***B**. Cul1 and nedd8 were detected in the nucleus in G0-synchronized NT2 cells. *The space bar is 5 μm.***C**. Cul4Bs did not colocalize with nedd8 *in vivo*. Human brain hippocampus was stained with the anti-Cul4B and mouse anti-nedd8. The image was taken near SVZ. A tiny amount of punctate nedd8 (white arrows) was also observed and appeared to represent neddylated Cul4B-3. A huge amount of unneddylated Cul4B was found in some cells that had DNA less accessible by DAPI. *The space bar is 10 μm. All images were obtained with a 40x objective.*

The above experiments suggest that the anti-Cul4B mainly recognized unneddylated Cul4B isoforms in brain tissues. We have shown previously that neddylated proteins are present in the nucleus of normal neuronal cells [34]. Cul4B sequences are highly conserved among mammalian species. Like rodent cells, human cells also have three major isoforms of Cul4B according to UniProtKB/Swiss-Prot data base. In human cells, compared to the canonical Cul4B-1 that has 913 amino acids, Cul4B-2 misses the first 22 amino acids and Cul4B-3 misses the first 196 amino acids and differs in amino acids 197-203. To determine if the anti-Cul4B mainly recognized unneddylated Cul4B, human NT-2 cells were immunostained with the rabbit anti-Cul4B and the mouse anti-nedd8 antibodies. In the metaphase cell, most Cul4B did not co-localize with nedd8 (Figure 2A, *top*). In this cell, Cul4B was present in the periphery whereas nedd8 accumulated around the condensed DNA. In old NT-2 cells with extended processes that suggested cell differentiation, the processes often had Cul4B especially at the process tip, but such Cul4B was not neddylated (Figure 2A, *middle*). In G0-synchronized NT-2 cells, nedd8 accumulated in the nucleus whereas Cul4B was still in the process tip (Figure 2A, *bottom*). In contrast to Cul4B, Cul1 was present in the nucleus in G0-synchronized NT-2 cells (Figure 2B).

To determine if Cul4B and nedd8 colocalized *in vivo*, human hippocampal brain sections were immunostained with the anti-Cul4B and the mouse anti-nedd8 antibodies (Figure 2C). Smaller cells and the ependymal cells along the ventricle were clearly labeled by the nedd8 antibody. However, the Cul4B-positive cells expressed little nedd8. A tiny amount of punctate nedd8 staining was present in the Cul4B-positive cells, which might represent neddylated Cul4B-3, but it was almost negligible compared to the huge amount of unneddylated Cul4B (Figure 2C). The huge amount of Cul4B appeared to inhibit DAPI staining of the DNA in these cells.

To summarize, the data showed that the anti-Cul4B specifically recognized Cul4B but not other Cullins. This antibody mainly recognized unneddylated Cul4B-1 and Cul4B-2 that accumulated in NPCs or NT-2 cells. Since all isoforms of Cul4B have the C-terminal neddylation consensus site, the data suggest that the presence of the extended N-terminus in Cul4B-1 and Cul4B-2 but missing in Cul4B-3 may inhibit neddylation.

### Cul4B was expressed during mitosis of the cell cycle in NPCs

**Figure 3:**
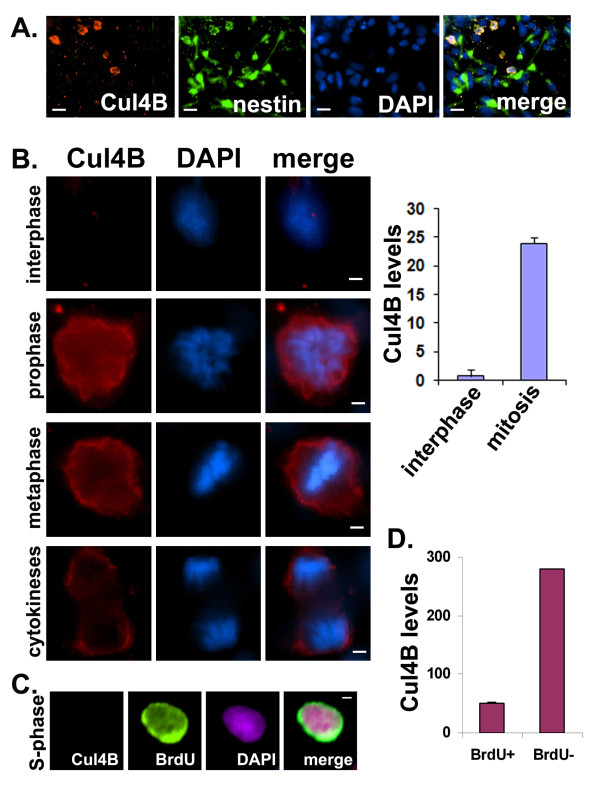
**Cul4B accumulated during mitosis of the cell cycle in neural progenitor cells (NPCs)*in vitro.*A**. Cul4B and nestin double-positive NPCs were found in rat NPC cultures. *x20 objective; scale bars, 4 μm*. **B**. Cul4B accumulated only during mitosis of the cell cycle in rat NPCs. *x60 objective; scale bars, 2 μm*. An analyses of Cul4B density in NPCs during interphase versus mitosis revealed a significant difference (*p = 5 × 10*^*-10*^*,* two-tail *t*-Test). *Y-axis, Cul4B levels in arbitrary units.***C**. NPCs undergoing DNA synthesis (green) did not express Cul4B. Cells undergoing DNA synthesis was identified by pulse-BrdU labeling right before fixation. *x60 objective; scale bars, 2 μm*. **D**. Mouse NPCs were analyzed after BrdU labeling. A comparison of BrdU-positive (BrdU+) versus BrdU-negative (BrdU-) cells shows that Cul4B levels were significantly higher in BrdU-negative cells than in BrdU-positive cells (*p* = 0.00016, two-tail *t*-Test). *Y-axis, Cul4B levels in arbitrary units.*

We have previously demonstrated our ability to grow primary NPCs that express nestin and Ki67 from E18 rat cortices [35]. Taking advantage of these primary cultures, we first determined the expression pattern of Cul4B during the cell cycle. Only a small fraction of NPCs expressed Cul4B in a given NPC culture (Figure 3A). These Cul4B-positive cells were also nestin-positive, but unlike the other nestin-positive cells, they appeared to have retracted their processes, a feature of cells undergoing mitosis. We then examined the expression of Cul4B during the cell cycle. Based on the distinct morphologies of the DNA in the cell cycle, Cul4B was expressed mainly during mitosis in NPCs (Figure 3B). Cul4B began to accumulate at the beginning of mitosis and it disappeared after cytokinesis. The analyses of Cul4B levels also showed that Cul4B levels were significantly higher during mitosis than during interphase in NPCs (Figure 3B, *right*).

To further confirm that Cul4B was absent in interphase cells, NPCs were pulse-labeled with BrdU to identify the cells undergoing DNA synthesis. In this experiment, NPCs were exposed to BrdU for 15 min and fixed right after the labeling. The brief treatment with BrdU ensured that only those NPCs undergoing S-phase DNA synthesis would incorporated BrdU. Longer exposure with BrdU would label cells at the other stages of the cell cycle as they grew continuously. The experiment showed that Cul4B was not present in the BrdU-labeled S-phase cells in rat primary NPCs (Figure 3C). Similar BrdU-labeling was performed in NPCs isolated from Day-0 newborn mouse cortices. The analyses showed that BrdU-positive S-phase cells had significantly lower levels of Cul4B than BrdU-negative cells (Figure 3D). These data suggest that Cul4B plays a specific role in the progression of mitosis.

### Downregulation of Cul4B arrested NPCs in the G2/M phase of the cell cycle

**Figure 4:**
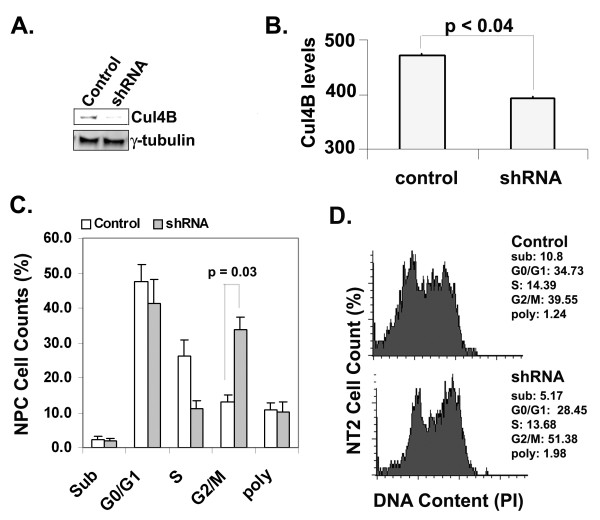
**Downregulation of Cul4B in NPCs or NT-2 cells led to cell cycle arrest at the G2/M phase.A**. Downregulation of Cul4Bs by shRNAs via HSV-1 vector suppressed Cul4B levels in NT-2 cells. The control expressed missense shRNA. *n = 3.***B**. Cul4B shRNA suppressed Cul4B expression in rat NPCs. NPCs transfected with the control vector or the Cul4BshRNA vector were identified by GFP expression. Images were collected in the same settings. Analyses of GFP-positive cells showed that Cul4BshRNA significantly downregulated Cul4B in NPCs (*p < 0.04*, t-Test). Density of Cul4B fluorescence is presented in the bar graph (Y-axis, arbitrary levels). **C**. Flow cytometric data analyses show that Cul4B downregulation by shRNAs arrested the cell cycle at the G2/M phase in NPCs. NPCs were transfected or infected with the shRNA vector (control) or the vector that had Cul4BshRNA. The graph represented data analyses from four independent experiments. *Control versus shRNA-expressing cells in the G2/M phase, two tail t-Test, p = 0.03.***D**. A representative histogram of NT-2 cells shows that downregulation of Cul4B by Cul4BshRNA via HSV-1 vector increased the number of cells in the G2/M phase of the cell cycle. A representative experiment is shown. *n = 3.* No increase in apoptotic cells (sub) was found due to Cul4BshRNA expression in either NT-2 cells or NPCs.

To examine the function of Cul4B during mitosis, a Cul4BshRNA vector was constructed to downregulate Cul4B expression. The selected targeting sequence (GATAAGCCTAAATTACCAGAA) was previously tested by another laboratory [36]. This sequence is present in all three isoforms of Cul4B. The expression of shRNAs is under the control of the tRNA^val^ promoter [37,38]. A GFP open-reading frame under the CMV promoter is also present in this vector. When packaged into HSV-1, the Cul4BshRNA vector efficiently downregulated Cul4B in NT-2 cells compared to the control missense shRNA (Figure 4A). This suggests that the Cul4B shRNA effectively suppressed the expression of total Cul4B.

The effect of the Cul4BshRNA vector in downregulating Cul4B in primary NPCs was then examined in transfected cells (based on GFP expression) by immunofluorescence microscopy. The analyses of these NPCs revealed that Cul4BshRNA significantly downregulated Cul4B compared to the control in NPCs (Figure 4B).

Since Cul4B was expressed during mitosis of the cell cycle, we next determined if Cul4B downregulation by shRNAs would affect cell cycle progression through the G2/M phase of the cell cycle. The DNA content was determined in the transfected or infected, GFP-positive NPCs by flow cytometry after propidium iodide staining (Figure 4C). The analyses showed that Cul4B shRNA-expression significantly increased the number of cells in the G2/M phase of the cell cycle. Similar trends were observed in NT-2 cells expressing Cul4BshRNAs compared to those expressing missense shRNAs (Figure 4D). Cul4B suppression by shRNAs and expression of GFP did not induce apoptosis since the number of apoptotic/sub-G0/G1 cells did not increase. These data suggest that Cul4B was necessary for cell cycle progression through the G2/M phase of the cell cycle in both NPCs and NT-2 cells.

### Cul4B-positive NPCs in nestin-GFP mice accumulated β-catenin

**Figure 5:**
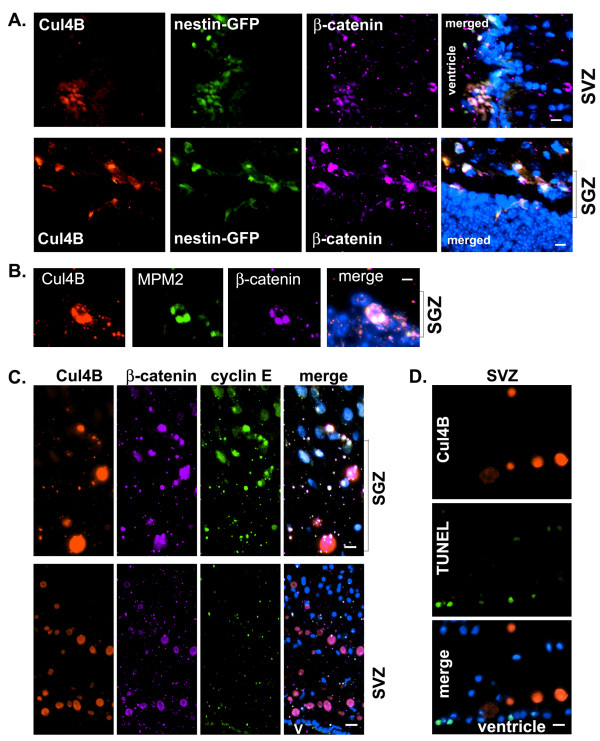
**Cul4B and β-catenin accumulated in the same NPCs*in vivo*in mouse and human brains.A**. Using the nestin-GFP mice, the subventricular zone (SVZ) and subgranular zone (SGZ) NPCs were identified by immunostaining with the mouse anti-GFP antibody. Cul4B-positive NPCs strongly expressed β-catenin. *x20 objective; scale bar, 15 μm*. **B**. Cul4B-expressing NPCs had the mitotic epitope as shown by the MPM-2 antibody-staining. *x40 objective; scale bar, 5 μm*. **C**. In normal human brains, Cul4B-positive cells accumulated β-catenin in both SGZ and SVZ, but cyclin E was only increased in SGZ in Cul4B-positive cells. **D**. TUNEL staining showed that Cul4B-positive cells did not have fragmented DNA although some other cells did have. *x20 objective in C and D; Blue, DAPI counterstain;* s*cale bar, 10 μm. SGZs are labeled with a bracket and a more compact layer of granule cells are visible for orientation.V, ventricle.*

Interestingly, β-catenin has been shown to accumulate periodically in the late G2/M phase of the cell cycle and abruptly decrease in the subsequent G1 phase in immortalized mouse epidermal keratinocyte MCA3D cells [39]. SVZ and SGZ are the major neurogenic niches in the brain. To evaluate the activity of Cul4B in NPCs *in vivo*, we used the nestin-GFP mouse strain that expressed GFP under the nestin-promoter, labeling all nestin-positive NPCs [40]. The brain sections were co-immunostained with the antibodies against Cul4B, GFP, and β-catenin. The results showed that some NPCs expressed high levels of both Cul4B and β-catenin in the SVZ and SGZ (Figure 5A & B). Since β-catenin levels were high in the same NPCs, the data suggest that the constitutive degradation of β-catenin was prevented in these NPCs in late mitosis *in vivo.* Although Cul4B-3 was neddylated, the data suggest that Cul4B-3 was unable to downregulate β-catenin probably due to its scanty presence in these cells. Alternatively, unneddylated Cul4Bs might play a role in stabilizing β-catenin by forming complexes that prevented Cul4B-3 ligase assembly and activation.

### Cul4B-positive cells expressed the mitotic marker MPM-2 *in vivo*

*In vitro* analyses suggest that Cul4B accumulation is necessary for NPC cell cycle progression through mitosis. In order to determine the cell cycle status of the NPCs *in vivo*, brain sections were immunostained with the MPM-2 monoclonal antibody. The MPM-2 antibody was originally raised against mitotic HeLa cells. It specifically recognizes a cell cycle-regulated, serine/threonine-phosphorylated epitope present in mitotic and meiotic proteins from a wide variety of species. These proteins become phosphorylated at the G2/M transition and are dephosphorylated at the end of mitosis [41]. In brain sections from one-month-old mice, Cul4B-positive cells were also stained with the MPM-2 antibody in SGZ (Figure 5B). These data further support the observations that unneddylated Cul4B accumulated during mitosis.

### Cul4B-positive NPCs in human brain accumulated β-catenin and were not apoptotic

As shown in Figure 2C, some cells in the human brain expressed a huge amount of Cul4B that co-expressed little nedd8. We found that these Cul4B-positive cells were mainly located in SVZ and SGZ, two major neurogenic areas of the human brain (Figure 5C). In these cells, β-catenin levels were very high. The accumulation of both Cul4B and β-catenin in the same cells again suggests that Cul4B did not downregulate β-catenin during mitosis, but instead Cul4B might have stabilized β-catenin. In the same cells, cyclin E only accumulated in NPCs in SGZ, not in SVZ, suggesting that Cul4B might regulate cyclin E only in SGZ.

The *in vivo* data suggest that the accumulation of unneddylated Cul4B during mitosis in NPCs is a physiological phenomenon. We next determined if these Cul4B-positive cells were healthy and not undergoing apoptosis by TUNEL staining. As shown in Figure 5D, cells that expressed Cul4B were negative for TUNEL staining. In contrast, some nearby Cul4B-negative ependymal cells lining the ventricle were TUNEL-positive. Thus, the Cul4B-positive cells did not have fragmented DNA, indicating that they were not apoptotic. Together, these data suggest that the accumulation of Cul4B during mitosis plays a physiological role in NPC cell cycle progression in the human brain.

## Discussion

Since Cul4B mutations are associated with XLID, this study examined the functions of the endogenous Cul4B in NPCs and brain tissues. Through a series of immunoprecipitation-western experiments, this study first demonstrated that the anti-Cul4B antibody specifically detected Cul4B, especially the two larger isoforms Cul4B-1 and Cul4B-2. Further analyses demonstrated that the two larger isoforms were unneddylated whereas the smaller isoform Cul4B-3 was neddylated in rodent brain protein extracts. This study showed that downregulation of Cul4B in rodent NPCs or human NT-2 cells arrested the cell cycle in the G2/M phase, suggesting that Cul4B accumulation was necessary for cell cycle progression through mitosis in these cells. Furthermore, Cul4B-positive cells co-expressed the MPM-2 mitotic epitope in brain sections, suggesting that Cul4B also accumulated *in vivo* in NPCs during mitosis. Finally, Cul4B expression was not associated with DNA fragmentation *in vivo*. Together, these data suggest that a physiological function of Cul4B is in mitosis progression in NPCs, which involves the accumulation of inactive Cul4B during mitosis.

Neddylation serves as a switch for activating CRL activity. This study demonstrated that the two larger isoforms of Cul4B, Cul4B-1 and Cul4B-2, were not neddylated, which suggest that these two isoforms of Cul4B may be inactive in downregulating substrates. The absence of neddylation in Cul4B-1 and Cul4B-2 was not caused by spontaneous deneddylation since the inhibitor, OPT, was present in the experiment. The anti-nedd8 antibody also worked since it recognized the smaller Cul4B-3 precipitated by the Cul4B antibody. A recent study has shown that more Cul4B is constitutively associated with the deneddylase COP9 than Cul1 [33]. Such tight association of Cul4B with COP9 may account for the absence of neddylation in Cul4B-1 and Cul4B-2 isoforms, which we found were more abundant in brain tissues. Despite the absence of neddylation in Cul4B-1 and Cul4B-2, Cul4B-3 that lacked the N-terminal portion was neddylated. In NT-2 cells and brain tissues, DDB1 was below the detection limit in whole protein extract. The anti-Cul4B antibody precipitated very little DDB1, which on one hand suggest that most Cul4B was not assembled with DDB1 and on the other suggests that the co-precipitated DDB1 was probably bound to Cul4B-3. Since the major difference between Cul4B-3 and the other two isoforms was the lack of the N-terminal sequences in Cul4B-3, the data suggest that the N-terminus of Cul4B may inhibit neddylation which normally occurs in the consensus lysine residue in the C-terminal Cullin domain. During the preparation of this manuscript, at least two Cul4B-knockout mouse models have been attempted but both resulted in early embryonic lethality [42,43]. Human mutations include C-terminal truncations or misssense mutations, which are predicted to affect Cul4B ubiquitin ligase activity, but null mutations are not observed. These observations suggest the N-terminus of Cul4B may support survival of human mutation carriers by a mechanism involving inhibition of neddylation.

This study also showed that increased levels of Cul4B co-existed with high levels of β-catenin in NPCs in the neurogenic niches, SGZ and SVZ, from both rodent and human brains. Neddylation is necessary for β-catenin degradation since β-catenin accumulates in NPCs expressing a dominant negative nedd8-conjugating enzyme Ubc12 (Chen unpublished data). There was also little evidence that the Cul4B-expressing NPCs co-expressed any nedd8. These data suggest that unneddylated Cul4B accumulated in a particular population of NPCs *in vivo*. Previous studies have shown that in the absence of Wnt signaling, β-catenin is constitutively downregulated via the SCF complex [44]. Furthermore, β-catenin may be a Cul4B ligase substrate [28,29] although it has not been replicated by independent laboratories. Together with the findings in this study, the data suggest that Cul4B-1 and Cul4B-2 may compete with neddylated Cul1 and possibly neddylated Cul4B-3, to transiently inhibit the degradation of certain substrates such as β-catenin during mitosis. In this sense, the unneddylated Cul4B still plays an active role in mitosis progression. It has been shown that β-catenin peaks periodically at the G2/M phase of the cell cycle in proliferating cells [45,46]. A specific function of β-catenin has been shown to establish the bipolar mitotic spindle during mitosis [47]. These previous studies suggest that β-catenin is specifically protected from degradation during mitosis, which may involve the accumulation of unneddylated Cul4Bs. Future studies may focus on isoform-specific effects of Cul4B in β-catenin downregulation when isoform-specific Cul4BshRNAs and antibodies are available.

Why does Cul4BshRNAs arrest NPCs in G2/M phase of the cell cycle? The Cul4BshRNAs used in this study targeted a sequence present in all three isoforms, the overall effect of which was to cause cell cycle arrest. Cul4B is clearly necessary in early embryonic development since Cul4B null mice have an embryonic lethal phenotype [42,43], which suggests that it functions in cell cycle progression. The current study suggests that Cul4B not only functions in mitosis progression but also in cell cycle withdrawal. In proliferating NPCs and NT-2 cells, maybe all isoforms of Cul4B are necessary for cell cycle progression, whereas in differentiating cells, Cul4B-1 and Cul4B-2 may play a major role. NPCs in human brains had a huge amount of Cul4B in the cell body but very little nedd8, which suggests that unneddylated Cul4Bs probably together with the small amount of neddylated Cul4B-3, are necessary for mitosis progression. The major supporting evidence for a role of Cul4B in cell cycle withdrawal is that unneddylated Cul4Bs were abundant in growing processes in differentiating or G0-synchronized NT-2 cells. Future investigations may focus on the potential isoform-specific effects of Cul4B.

This study did not detect Cul4B in S-phase NPCs and downregulation of Cul4B caused cell cycle arrest in G2/M in these cells. This is in contrast to the findings in HeLa cells where Cul4B knockdown results in S-phase accumulation [27]. In addition, another study shows that silencing of Cul4B in primary MEF cells has little effect on cell proliferation [21]. The discrepancies may be due to cell type and methodology differences. The current study mainly employed primary NPCs and examined endogenous Cul4Bs. The BrdU labeling in the current study lasted for only 15 min. Previous studies have also shown that cyclin E is a Cul4B substrate, but it remains controversial given the stimulatory role of cyclin E in S phase progression and cell proliferation. The data from the current study also raised questions whether Cul4B regulates cyclin E in SVZ NPCs. The differential neddylation of Cul4B isoforms as well as lack of specific antibodies as we found in this study suggest that caution is needed in interpreting the available data.

## Conclusions

This study provides strong evidence that the accumulation of Cul4B during mitosis is necessary for NPC cell cycle progression through mitosis. The data suggest that unneddylated Cul4B isoforms specifically inhibit β-catenin degradation during mitosis. The data also show that Cul4B-3 that misses the N-terminus present in the other two isoforms is neddylated, suggesting that the N-terminus may inhibit neddylation in the larger isoforms. Furthermore, the presence of unneddylated Cul4B in NT-2 cell processes suggests that it may have functions other than mitosis. The huge amount of unneddylated Cul4B in human brain hippocampal NPCs further suggests that Cul4B plays a pivotal role in brain development. These findings have opened new avenues for future investigations, which will help understand the cognitive deficits present in Cul4B-linked XLID.

## Methods

### Antibodies

Primary antibodies used in this study include rabbit anti-Cul4B (cat# 12916-1-AP) and rabbit anti-DDB1 (Proteintech Group, Chicago, USA), mouse anti-nestin (BD Bioscience, Sparks, MD, USA.), rabbit anti-β-catenin and mouse anti-nedd8 (Sigma), rabbit anti-nedd8 (Zymed), mouse anti-GFP (Antibody Inc.), mouse anti-MPM-2 (Millipore), rabbit anti-Cul4A (Dr. Kristin T. Chun), rabbit-anti-Cul1 (Neomarker), goat anti-β-catenin (sc-1496), and mouse anti-cyclin E (sc-25303) (the last two were from Santa Cruz Biotechnology, Santa Cruz, CA, USA). The secondary antibodies conjugated to Alexa fluor 488, Alexa fluor 564, and Alexa fluor 680 were obtained from Invitrogen (Calsbad, CA, USA).

### Construct

To construct the GFP-Cul4B(1-173) fusion protein (GFP173), the N-terminal fragment of Cul4B (1-173) was first produced by PCR fragment using human Cul4B as the template (Open Biosystems). This fragment was then fused to the C-terminus of GFP and cloned into the EcoRV/Kpn1-digested pcDNA3.1/Zeo- vector (Invitrogen). The clone was digested and sequenced for verification. The DNA was transfected into primary NPCs using Lipofectamine LTX (Invitrogen) according to manufacturer’s protocols.

### Immunoprecipitation and western blotting using fresh mouse brain tissues

Mouse brain tissues were harvested from adult C57BL/6 mice (Charles River Laboratories International). Immunoprecipitation and western blotting were performed essentially as described before [37]. Briefly, freshly harvested mouse brains were homogenized in IP buffer containing 20 mM Tris (pH 8), 1 mM MgCl2, 125 mM NaCl, 2% CHAPS, 10% glycerol, and a mixture of protease and phosphotase inhibitors including 5 μg/ml of the proteasome inhibitor MG132 and 1,10-orthophenathroline (OPT, 2 mM) [48]. The homogenate was precleared by centrifugation and protein G sepharose 4B beads before it was incubated with the primary antibody prebound to protein G sepharose 4B. The control was incubated with protein G sepharose 4B without the antibody. After the immune complex was resolved on an 8-16% precise protein gel (Pierce), the protein was transferred to nitrocellulose and blocked for 1h with 3% BSA. The molecular weight of Cul4B proteins were as reported in Uniprot and identified by comparing with the Precision Plus protein standards (BioRad) on the same blot.

### Primary NPC cultures

Primary NPCs were harvested from rat embryonic day 18 (E18) cerebral cortices from timed-pregnant Sprague Dawley rats (Charles River Laboratories International, Wilmington MA, USA). Animals were dissected according to the protocols approved by the institutional animal care committee. NPCs were grown as described before [35]. Briefly, cells were initially plated as monolayers on poly-ornithine and fibronectin-coated plates. Cells were then used at the first or second passage after the initial plating for flow cytometry. For cells used for immuno-staining, the cells were initially plated to grow neurospheres in non-coated dishes or monolayer in coated dishes. The cells were subsequently plated on coated glass-coverslips for analysis by immunostaining. In some cases as identified in the context, primary NPCs were grown from Day-0 C57BL/6 (Charles River) cortices by a similar method. The only difference was that mouse cortical tissue was digested with Trypsin for 1 h before centrifugation and plating.

### NPC immunostaining

NPCs were fixed in 4% paraformaldehyde for 20 min at room temperature. After 3 washes in PBS, cells were treated with 2N HCl for 30 min at room temperature. Cells were then washed and incubated in the blocking buffer (5% BSA or normal goat serum/0.5% Triton X-100/10 mM PBS, pH 7.4) for 15 min. Cells were incubated with primary antibodies for 2 h, washed, and then incubated with secondary antibodies for 1 h. At the end of the antibody incubation, cells were stained with DAPI, rinsed 5x, and mounted in 90% glycerol/PBS. Images were taken using a 20x or 60x objective by a Nikon Eclipse 600 or Nikon 90i epifluorescence microscope and the program NIS-elements (Nikon). The intensity of staining was identified by the NIS-element software. NT-2 cells were processed similarly.

### BrdU labeling of NPCs and analyses

Proliferating rat (from E18 cortices) or mouse (from Day 0 cortices) NPCs grown on coated glass coverslips were incubated with BrdU (50 uM) for 15 min and fixed in 4% paraformaldehyde for 40 min. Cells were then immunostained as described above. The analyses of Cul4B expression in BrdU-positive versus BrdU-negative mouse NPCs were carried out using the NIS-element software based on average density of Cul4B, which subtracted the average background noise obtained in the same images. The data were analyzed by *t*-Test assuming unequal variance (Microsoft Excel data analyses tool).

### Cul4B shRNA design and downregulation in NPCs

The Cul4B shRNA targeting sequence “gataagcctaaattaccagaa” was designed based on a previously published siRNA sequence [36]. The targeting sequence is present in all three isoforms of Cul4B. It was constructed in the shRNA vector pHSVGET that we designed before [37,38]. The difference between this protocol and our previous one was that in the current study NPCs were transfected with the vector that was not packaged into a recombinant HVS-1 virus. The pHSVGET vector allowed the expression of the shRNA under the tRNA^val^ promoter and the GFP protein under the CMV promoter after the transfection. Since the transfection efficiency was relatively low, the levels of Cul4B in the GFP-positive cells were determined by analyzing the microscopic images after they were taken at the same settings using a Nikon Eclipse 600 epifluorescence microscope and the program NIS-elements (Nikon). The expression levels of Cul4B in 38 (control) and 47 cells (Cul4BshRNA) were averaged and analyzed by one tail *t*-Test using Excel (Microsoft).

Besides transfection, the shRNA vectors were also packaged into HSV-1 for efficient downregulation in NT-2 cells (ATCC) and in primary NPCs. Efficient knockdown of Cul4B was achieved with 0.5 infectious unit per cell [37,38].

### Flow cytometric data analyses

After NPCs were transfected for 20 h, cells were fixed in 4% paraformaldehyde for 20 min at room temperature. Samples were washed twice in PBS and then incubated in a buffer containing 0.02% NP40 for 1.5 h on ice while cells were being counted. Alternatively, cells were fixed in 70% ethanol for 5 h on ice, which we found would not affect GFP fluorescence. After two washes in PBS, cells were incubated with 100 μg/ml RNase A for 30 min at 37^o^C. Cells were then incubated overnight in a buffer containing 10 μg/ml propidium iodide and 10 μg/ml RNase A in PBS. The amount of the solution was adjusted in proportion to the amount of cells in each sample. Cells were washed twice before they were subjected to flow cytometry analyses with a FACSCalibur flow cytometer (Becton, Dickinson and Company, Franklin Lakes, NJ, USA) equipped with an argon ion laser (488 nm) for the analyses of GFP-expressing cells and a laser with an excitation at 635 nm for PI-labeled DNA content analyses. Transfected GFP^+^ cells were gated and compared in their DNA content after propidium iodide staining using the software ModFit LT (Version 3.0, Verity Software House, Topsham, ME, USA) as described before [35]. Around 300 to 800 GFP-positive cells were analyzed in these experiments. The experiment was repeated using HSV-1-mediated expression of shRNAs in NT-2 cells and NPCs, in which case, more than 5,000 GFP-positive cells were analyzed.

The data were analyzed by *t*-Test assuming unequal variance (Microsoft Excel data analyses tool). NPC analyses were based on four independent experiments with two from rat NPCs and two from mouse NPCs. The analyses of NT-2 cells were based on three independent experiments.

### Nestin-GFP mice

The nestin-GFP transgenic mice were as previously developed and characterized [40]. The brain tissue was harvested from one month-old mice, fixed in 4% paraformaldehyde, processed for paraffin-embedding, and sectioned in 6μm-thick sections. After dewaxing and rehydration, sections were incubated in 2N HCl for 30 min. Sections were then washed twice in distilled H_2_O and once in PBS, and blocked and permeablized in Blocking Buffer (5% BSA/1% Triton X-100/PBS, pH 7.4) for 15 min. Sections were incubated in primary antibodies diluted in 5% BSA/0.05% Triton X-100/PBS for 2 h. After being washed twice in 0.05% Triton, sections were incubated with the secondary antibodies diluted in Blocking Buffer for 1 h. Slides were immediately stained with DAPI (10 ug/ml) for 10 min. After washing once in 0.05% Triton, and three times in PBS, slides were coverslipped with 90% glycerol. The sections incubated in the same buffers without the primary antibodies were used as negative controls. Images were collected with an x40 objective using a Nikon Eclipse 600 epifluorescence microscope.

### Human brain tissue immunostaining

Pre-characterized and paraffin-embedded blocks of hippocampus from human brain samples were obtained from Harvard Brain Bank. The postmortem brain tissues were collected for research purposes with the approval by Harvard research ethics committee based on NIH guidelines and in compliance with the Helsinki Declaration. After paraffin-embedded hippocampal blocks were sectioned at 6 μm thickness. After dewaxing and rehydration, the sections were incubated in 2N HCl for 30 min. The sections were then washed and blocked in Blocking Buffer for 15 min. The sections were incubated in primary antibodies diluted with the Blocking Buffer for 2 h. After two washes in 0.1% Triton, the sections were incubated with the secondary antibodies diluted in Blocking Buffer for 1 h. The slides were immediately stained with DAPI. To block lipofoscin, the slides were incubated with 0.3% Sudan Black in 70% ethanol for 6 min. After washing twice in 0.5% Triton and 7x in PBS, the slides were coverslipped with 90% glycerol. The sections incubated in the same buffers without the primary antibodies were used as negative controls. Images were collected with an x20 objective using a Nikon Eclipse 600 epifluorescence microscope.

### TUNEL staining for DNA fragmentation

The human brain sections were processed for TUNEL staining using a modified protocol of the ApopTag Fluorescein In Situ Apoptosis Detection Kit (S7110, Millipore). Briefly, after dewaxing, rehydration, HCl-treatment, and blocking as described above, the brain sections were equilibrated with the Equilibration Buffer and treated with the TdT enzyme. Right after the enzyme reaction was stopped with the Stop/Wash Buffer, the section was rinsed with PBS and incubated with the anti-Cul4 antibody for 2 h at RT. After washes, the section was incubated with the fluorescein anti-digoxigenin conjugate (provided in the kit) and Alexa 594 goat anti-rabbit (Invitrogen) for 1 h. Samples were then stained with DAPI and Sudan Black as described above. A negative control was performed without the TdT enzyme and the primary antibody.

## Abbreviations

NPC: Neural progenitor cell; CRL: Cullin-RING ubiquitin ligases; OPT: 1, 10-orthophenathroline; RING: *r*eally *in*teresting *g*ene; SGZ: Subgranular zone; SVZ: Subventricular zone; XLID: X-linked intellectual disability.

## Competing interests

The authors declare no competing interests.

## Authors’ contributions

HCL and YC performed the experiments. YC designed the experiments and wrote the manuscript. GE helped with the nestin-GFP mice. All authors read and approved the final manuscript.

## References

[B1] Kishino T Lalande M Wagstaff J UBE3A/E6-AP mutations cause Angelman syndrome Nat Genet 1997 15 1 70 73 10.1038/ng0197-70 8988171

[B2] Matsuura T Sutcliffe JS Fang P Galjaard RJ Jiang YH Benton CS Rommens JM Beaudet AL De novo truncating mutations in E6-AP ubiquitin-protein ligase gene (UBE3A) in Angelman syndrome Nat Genet 1997 15 1 74 77 8988172 10.1038/ng0197-74

[B3] Greer PL Hanayama R Bloodgood BL Mardinly AR Lipton DM Flavell SW Kim TK Griffith EC Waldon Z Maehr R The Angelman Syndrome protein Ube3A regulates synapse development by ubiquitinating arc Cell 2010 140 5 704 716 10.1016/j.cell.2010.01.026 20211139 PMC2843143

[B4] Matsumine H Saito M Shimoda-Matsubayashi S Tanaka H Ishikawa A Nakagawa-Hattori Y Yokochi M Kobayashi T Igarashi S Takano H Localization of a gene for an autosomal recessive form of juvenile Parkinsonism to chromosome 6q25.2–27 Am J Hum Genet 1997 60 3 588 596 9042918 PMC1712507

[B5] Narendra D Tanaka A Suen DF Youle RJ Parkin is recruited selectively to impaired mitochondria and promotes their autophagy J Cell Biol 2008 183 5 795 803 10.1083/jcb.200809125 19029340 PMC2592826

[B6] Cabezas DA Slaugh R Abidi F Arena JF Stevenson RE Schwartz CE Lubs HA A new X linked mental retardation (XLMR) syndrome with short stature, small testes, muscle wasting, and tremor localises to Xq24-q25 J Med Genet 2000 37 9 663 668 10.1136/jmg.37.9.663 10978355 PMC1734699

[B7] Zou Y Liu Q Chen B Zhang X Guo C Zhou H Li J Gao G Guo Y Yan C Mutation in CUL4B, Which Encodes a Member of Cullin-RING Ubiquitin Ligase Complex, Causes X-Linked Mental Retardation Am J Hum Genet 2007 80 3 561 566 10.1086/512489 17273978 PMC1821105

[B8] Tarpey PS Raymond FL O'Meara S Edkins S Teague J Butler A Dicks E Stevens C Tofts C Avis T Mutations in CUL4B, which encodes a ubiquitin E3 ligase subunit, cause an X-linked mental retardation syndrome associated with aggressive outbursts, seizures, relative macrocephaly, central obesity, hypogonadism, pes cavus, and tremor Am J Hum Genet 2007 80 2 345 352 10.1086/511134 17236139 PMC1785336

[B9] Petroski MD Deshaies RJ Function and regulation of cullin-RING ubiquitin ligases Nat Rev Mol Cell Biol 2005 6 1 9 20 15688063 10.1038/nrm1547

[B10] Chen Y Neve RL Liu H Neddylation Dysfunction in Alzheimer's Disease J Cell Mol Med 2012 10.1111/j.1582-4934.2012.01604.x 10.1111/j.1582-4934.2012.01604.x PMC3484225 22805479

[B11] Soucy TA Dick LR Smith PG Milhollen MA Brownell JE The NEDD8 conjugation pathway and its relevance in cancer biology and therapy Genes Cancer 2010 1 7 708 716 10.1177/1947601910382898 21779466 PMC3092238

[B12] Duda DM Borg LA Scott DC Hunt HW Hammel M Schulman BA Structural insights into NEDD8 activation of cullin-RING ligases: conformational control of conjugation Cell 2008 134 6 995 1006 10.1016/j.cell.2008.07.022 18805092 PMC2628631

[B13] Saha A Deshaies RJ Multimodal activation of the ubiquitin ligase SCF by Nedd8 conjugation Mol Cell 2008 32 1 21 31 10.1016/j.molcel.2008.08.021 18851830 PMC2644375

[B14] Yamoah K Oashi T Sarikas A Gazdoiu S Osman R Pan ZQ Autoinhibitory regulation of SCF-mediated ubiquitination by human cullin 1's C-terminal tail Proc Natl Acad Sci USA 2008 105 34 12230 12235 10.1073/pnas.0806155105 18723677 PMC2519045

[B15] Bosu DR Kipreos ET Cullin-RING ubiquitin ligases: global regulation and activation cycles Cell Div 2008 3 1 7 10.1186/1747-1028-3-7 18282298 PMC2266742

[B16] Wei N Serino G Deng XW The COP9 signalosome: more than a protease Trends Biochem Sci 2008 33 592 600 10.1016/j.tibs.2008.09.004 18926707

[B17] Bosu DR Feng H Min K Kim Y Wallenfang MR Kipreos ET C. elegans CAND-1 regulates cullin neddylation, cell proliferation and morphogenesis in specific tissues Dev Biol 2010 346 113 126 10.1016/j.ydbio.2010.07.020 20659444 PMC2955628

[B18] Chen Y Liu W Naumovski L Neve RL ASPP2 inhibits APP-BP1-mediated NEDD8 conjugation to cullin-1 and decreases APP-BP1-induced cell proliferation and neuronal apoptosis J Neurochem 2003 85 3 801 809 10.1046/j.1471-4159.2003.01727.x 12694406

[B19] Higa LA Zhang H Stealing the spotlight: CUL4-DDB1 ubiquitin ligase docks WD40-repeat proteins to destroy Cell Div 2007 2 1 5 10.1186/1747-1028-2-5 17280619 PMC1805432

[B20] Li B Jia N Kapur R Chun KT Cul4A targets p27 for degradation and regulates proliferation, cell cycle exit, and differentiation during erythropoiesis Blood 2006 107 11 4291 4299 10.1182/blood-2005-08-3349 16467204 PMC1895787

[B21] Liu L Lee S Zhang J Peters SB Hannah J Zhang Y Yin Y Koff A Ma L Zhou P CUL4A abrogation augments DNA damage response and protection against skin carcinogenesis Mol Cell 2009 34 4 451 460 10.1016/j.molcel.2009.04.020 19481525 PMC2722740

[B22] Wang H Zhai L Xu J Joo HY Jackson S Erdjument-Bromage H Tempst P Xiong Y Zhang Y Histone H3 and H4 ubiquitylation by the CUL4-DDB-ROC1 ubiquitin ligase facilitates cellular response to DNA damage Mol Cell 2006 22 3 383 394 10.1016/j.molcel.2006.03.035 16678110

[B23] Guerrero-Santoro J Kapetanaki MG Hsieh CL Gorbachinsky I Levine AS Rapic-Otrin V The cullin 4B-based UV-damaged DNA-binding protein ligase binds to UV-damaged chromatin and ubiquitinates histone H2A Cancer Res 2008 68 13 5014 5022 10.1158/0008-5472.CAN-07-6162 18593899

[B24] Kerzendorfer C Whibley A Carpenter G Outwin E Chiang SC Turner G Schwartz C El-Khamisy S Raymond FL O'Driscoll M Mutations in Cullin 4B result in a human syndrome associated with increased camptothecin-induced topoisomerase I-dependent DNA breaks Hum Mol Genet 2010 19 7 1324 1334 10.1093/hmg/ddq008 20064923 PMC2838540

[B25] Ohtake F Baba A Takada I Okada M Iwasaki K Miki H Takahashi S Kouzmenko A Nohara K Chiba T Dioxin receptor is a ligand-dependent E3 ubiquitin ligase Nature 2007 446 7135 562 566 10.1038/nature05683 17392787

[B26] Higa LA Yang X Zheng J Banks D Wu M Ghosh P Sun H Zhang H Involvement of CUL4 Ubiquitin E3 ligases in Regulating CDK Inhibitors Dacapo/p27(Kip1) and Cyclin E Degradation Cell Cycle 2006 5 1 71 77 10.4161/cc.5.1.2266 16322693

[B27] Zou Y Mi J Cui J Lu D Zhang X Guo C Gao G Liu Q Chen B Shao C Characterization of nuclear localization signal in the N terminus of CUL4B and its essential role in cyclin E degradation and cell cycle progression J Biol Chem 2009 284 48 33320 33332 10.1074/jbc.M109.050427 19801544 PMC2785175

[B28] Tripathi R Sastry KS Kota SK Srinivas UK Cloning and characterization of mouse cullin4B/E3 ubiquitin ligase J Biosci 2005 30 3 329 337 10.1007/BF02703670 16052071

[B29] Tripathi R Kota SK Srinivas UK Cullin4B/E3-ubiquitin ligase negatively regulates beta-catenin J Biosci 2007 32 6 1133 1138 17954973 10.1007/s12038-007-0114-0

[B30] Nakagawa T Xiong Y X-Linked Mental Retardation Gene CUL4B Targets Ubiquitylation of H3K4 Methyltransferase Component WDR5 and Regulates Neuronal Gene Expression Mol Cell 2011 43 3 381 391 10.1016/j.molcel.2011.05.033 21816345 PMC3230935

[B31] Li X Lu D He F Zhou H Liu Q Wang Y Shao C Gong Y CUL4B ubiquitin ligase targets peroxiredoxin III for degradation J Biol Chem 2011 286 32344 32354 10.1074/jbc.M111.249003 21795677 PMC3173229

[B32] Hori T Osaka F Chiba T Miyamoto C Okabayashi K Shimbara N Kato S Tanaka K Covalent modification of all members of human cullin family proteins by NEDD8 Oncogene 1999 18 48 6829 6834 10.1038/sj.onc.1203093 10597293

[B33] Bennett EJ Rush J Gygi SP Harper JW Dynamics of cullin-RING ubiquitin ligase network revealed by systematic quantitative proteomics Cell 2010 143 6 951 965 10.1016/j.cell.2010.11.017 21145461 PMC3008586

[B34] Chen Y Liu W McPhie DL Hassinger L Neve RL APP-BP1 mediates APP-induced apoptosis and DNA synthesis and is increased in Alzheimer's disease brain J Cell Biol 2003 163 1 27 33 10.1083/jcb.200304003 14557245 PMC2173435

[B35] Chen Y Dong C Abeta40 promotes neuronal cell fate in neural progenitor cells Cell Death Differ 2009 16 3 386 394 10.1038/cdd.2008.94 18566600

[B36] Higa LA Mihaylov IS Banks DP Zheng J Zhang H Radiation-mediated proteolysis of CDT1 by CUL4-ROC1 and CSN complexes constitutes a new checkpoint Nat Cell Biol 2003 5 11 1008 1015 10.1038/ncb1061 14578910

[B37] Chen Y Bodles AM McPhie DL Neve RL Mrak RE Griffin WS APP-BP1 inhibits Abeta42 levels by interacting with Presenilin-1 Mol Neurodegener 2007 2 3 10.1186/1750-1326-2-3 17286867 PMC1802080

[B38] Chen Y Bodles AM Amyloid precursor protein modulates beta-catenin degradation J Neuroinflammation 2007 4 1 29 10.1186/1742-2094-4-29 18070361 PMC2231348

[B39] Olmeda D Castel S Vilaro S Cano A Beta-catenin regulation during the cell cycle: implications in G2/M and apoptosis Mol Biol Cell 2003 14 7 2844 2860 10.1091/mbc.E03-01-0865 12857869 PMC165681

[B40] Mignone JL Kukekov V Chiang AS Steindler D Enikolopov G Neural stem and progenitor cells in nestin-GFP transgenic mice J Comp Neurol 2004 469 3 311 324 10.1002/cne.10964 14730584

[B41] Engle DB Doonan JH Morris NR Cell-cycle modulation of MPM-2-specific spindle pole body phosphorylation in Aspergillus nidulans Cell Motil Cytoskeleton 1988 10 3 434 437 3052873 10.1002/cm.970100310

[B42] Cox BJ Vollmer M Tamplin O Lu M Biechele S Gertsenstein M van Campenhout C Floss T Kuhn R Wurst W Phenotypic annotation of the mouse X chromosome Genome Res 2010 20 8 1154 1164 10.1101/gr.105106.110 20548051 PMC2909578

[B43] Jiang B Zhao W Yuan J Qian Y Sun W Zou Y Guo C Chen B Shao C Gong Y Lack of cul4b, an e3 ubiquitin ligase component, leads to embryonic lethality and abnormal placental development PLoS One 2012 7 5 e37070 10.1371/journal.pone.0037070 22606329 PMC3351389

[B44] Kimelman D Xu W Beta-Catenin destruction complex: insights and questions from a structural perspective Oncogene 2006 25 57 7482 7491 10.1038/sj.onc.1210055 17143292

[B45] Davidson G Niehrs C Emerging links between CDK cell cycle regulators and Wnt signaling Trends Cell Biol 2010 20 8 453 460 10.1016/j.tcb.2010.05.002 20627573

[B46] Orford K Orford CC Byers SW Exogenous expression of beta-catenin regulates contact inhibition, anchorage-independent growth, anoikis, and radiation-induced cell cycle arrest J Cell Biol 1999 146 4 855 868 10.1083/jcb.146.4.855 10459019 PMC2156133

[B47] Kaplan DD Meigs TE Kelly P Casey PJ Identification of a role for beta-catenin in the establishment of a bipolar mitotic spindle J Biol Chem 2004 279 12 10829 10832 10.1074/jbc.C400035200 14744872

[B48] Cope GA Suh GS Aravind L Schwarz SE Zipursky SL Koonin EV Deshaies RJ Role of predicted metalloprotease motif of Jab1/Csn5 in cleavage of Nedd8 from Cul1 Science 2002 298 5593 608 611 10.1126/science.1075901 12183637

